# Simultaneous onset of Crohn's disease and Psoriasis in a Multiple Sclerosis patient treated with Teriflunomide: A novel case report highlighting potential autoimmune interactions

**DOI:** 10.1016/j.heliyon.2024.e26195

**Published:** 2024-02-09

**Authors:** Masoud Ghiasian, Alireza Rastgoo Haghi, Shiva Borzouei, Rashed Bawand

**Affiliations:** a Department of Neuroimmunology, School of Medicine, Hamadan University of Medical Sciences, Hamadan, Islamic Republic of Iran; b Department of Pathology, School of Medicine, Hamadan University of Medical Sciences, Hamadan, Islamic Republic of Iran; c Department of Internal Medicine, School of Medicine, Hamadan University of Medical Sciences, Hamadan, Islamic Republic of Iran; d Department of General Medicine, School of Medicine, Hamadan University of Medical Sciences, Hamadan, Islamic Republic of Iran

**Keywords:** Relapsing-remitting multiple sclerosis, Teriflunomide, Disease-modifying therapy, Crohn's disease, Inflammatory bowel disease, Psoriasis, Case report

## Abstract

Teriflunomide (TFN) is an oral Disease-modifying therapy (DMT) widely used in the treatment of relapsing forms of Multiple Sclerosis (MS). Although TFN has demonstrated efficacy in reducing MS activity, recent evidence suggests a possible association between TFN and the onset of rare and severe medical conditions. We present a novel case report of a 47-year-old woman with a history of MS who developed concurrent Crohn's disease and Psoriasis following TFN treatment. This unique occurrence has not been previously documented in the literature. The patient experienced gastrointestinal symptoms and changes in nail color while on TFN. Colonoscopy and biopsy revealed crypt architectural distortion and lamina propria expansion, indicative of Crohn's disease, while dermatological evaluation suggested Psoriasis. Consequently, TFN was discontinued and switched to alternative therapy (Glatiramer acetate), and the patient underwent close observation and regular evaluations. Three months after stopping the TFN, the patient's nail lesions disappeared completely, her abdominal pain and diarrhea were resolved, and the follow-up colonoscopy was completely normal. In this regard, the association between MS, Inflammatory Bowel Disease (IBD), and Psoriasis has been reported in previous studies, with potential involvement of Th17 and IL-17 pathways. Although gastrointestinal side effects with TFN use are typically mild and transient, rare cases of TFN-induced IBD have been reported. Dermatological disorders, including Psoriasis, have also been linked to TFN use, with similarities to our case report. Further research and awareness are warranted to better understand the potential side effects and long-term implications of TFN in the management of MS.

## 1 Introduction Background

Teriflunomide (TFN) is an oral Disease-modifying therapy (DMT) utilized in the treatment of relapsing forms of Multiple Sclerosis (MS). TFN, the active form of Leflunomide, works by reversibly blocking dihydroorotate dehydrogenase (DHODH), a crucial enzyme involved in the de novo synthesis of pyrimidine, and through this way, the proliferation of immune cells involved in the inflammation that occurs in MS can be inhibited. This results in a reduction in the frequency and severity of relapses, as well as a slowing of disease progression. TFN was first approved by the FDA in 2012 and has since been widely used for the management of MS [1,2].

Common side effects of TFN include hair thinning, nausea, diarrhea, and elevated liver enzymes. But, these side effects are generally mild and manageable, and the benefits of TFN in reducing MS activity and improving quality of life are considered important for many MS patients [1]. Nevertheless, recent evidence suggests a possible link between TFN and the onset of some rare and severe medical conditions [3,4].

In this regard, we have reported a distinctive case of an MS patient who experienced a concurrent onset of Crohn's disease and Psoriasis after using TFN. This case represents a novel and unprecedented occurrence, as it has not been previously documented in the literature.

## 2 Case report

In this case study, we discuss a 47-year-old female patient with a 14-year history of MS. Her early symptoms included a band-like sensation across her thoracic region. Initial MRI scans identified multiple periventricular lesions and a thoracic lesion, with no Gadolinium enhancement observed, and the diagnosis of MS was established for her based on meeting the McDonald criteria. Despite the results of the diagnostic tests, the initiation of treatment was delayed for several years due to the patient's initial reluctance. The decision to start therapy came after she experienced a classic episode of optic neuritis, marked by unilateral blurred vision and pain. This diagnosis was supported by MRI findings showing enhanced optic nerve signal with Gadolinium contrast and high signal intensity on T2-weighted images. An ophthalmology consultation excluded alternative diagnoses like uveitis. The patient was treated with a course of methylprednisolone (1000 mg IV daily for 5 days, followed by a tapering dose of oral steroids), that effectively relieved her symptoms. Afterward, a DMT (Interferon beta-1a) was initiated for her, which was continued for four years, and during this time, periodical evaluations were performed, and the patient's results were satisfying, with her Expanded Disability Status Scale (EDSS) remaining ≤1.

**Fig. 1:**
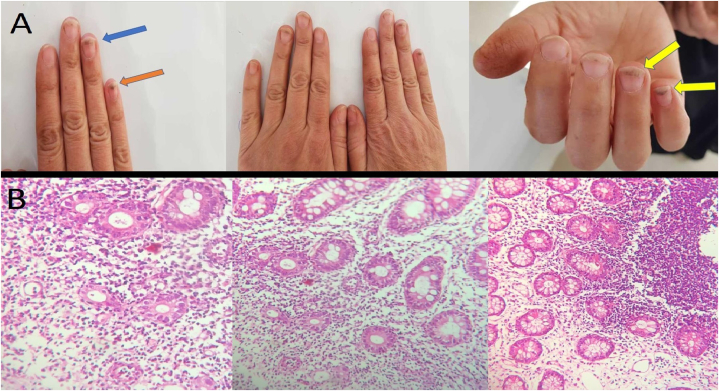
Simultaneous onset of Crohn's disease and Psoriasis in a Multiple Sclerosis patient treated with Teriflunomide; **A:** Macroscopic images of the patient's nail lesions, consistent with the clinical diagnosis of Psoriasis. **B:** Microscopic images of the patient's colon tissue section stained with hematoxylin and eosin (H & E), with the magnification of 400×: Sections revealing colonic mucosa with crypt architectural distortion & expansion of the lamina propria with mild to moderate mixed inflammatory cell infiltration and cryptitis, consistent with the diagnosis of Crohn's disease.

After four years of treatment, the patient experienced depression, unsuccessfully treated with a Selective Serotonin Reuptake Inhibitor (SSRI). As the result, her DMT was changed to TFN (14 mg PO qDay). By performing this change, the depressive symptoms improved significantly; but the patient developed mild diarrhea and abdominal pain. Given the patient's stable and regular dietary habits, absence of recent travel or intake of potentially risky foods, and lack of previous gastrointestinal disorders or recent antibiotic use, a symptomatic approach using Loperamide was initiated for treatment. The patient responded positively to this treatment, leading to the resolution of the gastrointestinal symptoms. Nonetheless, after a few weeks, her GI symptoms returned, and this time they were more severe, and did not respond to the previous treatments. Accordingly, a stool examination was conducted, along with serum tests for antibodies commonly associated with celiac disease (including anti-gliadin, anti-endomysial antibody, and anti-tissue transglutaminase). The results of the stool analysis indicated normal parameters, with no evidence of excess fat, blood, or indications of bacterial or parasitic infections. Furthermore, the tests for celiac-specific antibodies yielded negative results. Moreover, the patient complained about changes in the color of her nails (Fig. 1-A). Therefore, consultations with gastroenterology and dermatology services were requested.

The department of gastroenterology conducted a colonoscopy procedure on the patient, during which several macroscopic lesions were seen in the colon, and biopsies were taken from theses lesions. These biopsy samples were subsequently sent to the pathology laboratory for evaluation, which revealed crypt architectural distortion and expansion of the lamina propria with mild to moderate mixed inflammatory cell infiltration and cryptitis, indicative of Crohn's disease (Fig. 1-B). In addition, apart from this, the dermatology service suggested that the patient's nail changes were likely indicative of Psoriasis.

Subsequently, the patient's DMT (TFN) was discontinued. TFN demonstrates complete oral bioavailability (100%) and reaches peak plasma concentrations within 1–4 hours post-administration [5]. Nonetheless, its prolonged half-life of approximately 18–19 days, coupled with significant enterohepatic recirculation, results in about 37.5% of the orally administered dose being excreted unchanged in the feces even three weeks after ingestion [5]. In light of this, to accelerate the removal of TFN from the patient's system, we implemented a strategy involving cholestyramine. The patient received cholestyramine in a carefully calculated dosage, under stringent medical supervision, for a set period. This measure was taken to prevent any further aggravation of autoimmune conditions and to smoothly transition the patient to alternative therapeutic options. Following the cessation of TFN, the patient's DMT was switched to Glatiramer Acetate (GA). No additional treatments were introduced at this stage. Afterward, the patient was subjected to meticulous monitoring and routine assessments to ensure optimal management of her condition.

Three months after stopping the TFN, the patient's nail lesions disappeared completely, and her abdominal pain and diarrhea were resolved. A follow-up colonoscopy was performed, which was completely normal.

Moreover, after more than one and a half years, the patient was evaluated again and had no gastrointestinal or skin symptoms.

## 3 Discussion

The association between MS, Inflammatory Bowel Disease (IBD), and Psoriasis has been reported in various studies. Even, a systematic review and meta-analysis conducted by Dobson and Giovannoni in 2013 [6] declared a statistically significant association between MS and IBD (OR = 1.56; *P* < 0.0001), as well as between MS and Psoriasis (OR = 1.31; *P* < 0.0001). In this regard, Waite and Skokos [7] suggested that this association may originate from T helper 17 (Th17) and interleukin-17 (IL-17) pathway, and Toussirot [8] attributed this association to another cytokine of Th17s, which is IL-23. Furthermore, some other studies have implicated pathways associated with “Tumor Necrosing Factor-alpha” (TNF-α) in this correlation [9].

Although gastrointestinal side-effects in TFN users are commonly reported, but these complications are usually mild and transient and can be effectively managed with symptomatic treatment [1]. The patient in this study initially presented with mild abdominal pain and diarrhea, which resolved with Loperamide. However, there have been two reported cases [3,10] of TFN-induced IBD in the literature (Table 1). Therefore, healthcare professionals should be aware that while most TFN users may only experience mild and short-term gastrointestinal complications, severe complications such as IBD can occur in rare cases and should not be neglected.

**Table 1:** Review of the literature examining reported cases linking Teriflunomide to the development of IBD and Psoriasis.

	Report	Publish year	Country	Patient's age (Year)	Patient's Gender	Summary
**TFN & IBD**	Son et al. [10]	2020	UK	39	Female	A 39-year-old female with a history of depression and Hashimoto thyroiditis, developed intermittent paresthesias and was diagnosed with MS, experienced severe gastrointestinal symptoms shortly after starting treatment with Teriflunomide. Within days of initiating the therapy, she developed abdominal cramping and severe diarrhea, leading to a weight loss of 20 pounds over a few months and was later diagnosed with ***lymphocytic colitis***. She responded to treatment with Budesonide.
	Esfahani et al. [3]	2020	US	49	Male	A patient with Psoriasis, asthma, and MS that treated with Teriflunomide developed severe diarrhea and unintentional weight loss. Upper GI endoscopy showed non-bleeding gastric and duodenal ulcers, and colonoscopy revealed histopathological findings suggestive of ***Crohn's disease***. He achieved clinical remission with Vedolizumab but switched to Ustekinumab due to intolerance.
**TFN & Psoriasis**	Couper & Shaffrali [19]	2022	UK	52	Male	A 52-year-old man with MS received Teriflunomide, experiencing skin issues within three days, including ***pustules, red scaly plaques***, and edema on his palms and feet. Initial treatments involved topical hydrocortisone and emollient, followed by antibiotics and a topical corticosteroid with limited benefit. Dermatology review led to additional treatments, and after six weeks, psoriatic ***nail disease*** developed. Treatment with calcipotriol ointment and acitretin resulted in significant improvement, and a three-month course of acitretin was completed successfully.
	Negrotto & Correale [16]	2019	Argentina	52	Female	An MS patient stopped subcutaneous interferon beta1a due to injection-related pain and later developed left leg weakness and a ‘band-like’ sensation on the left abdomen. Teriflunomide was started, but a month later she developed painful bilateral ***palmar pustular lesions diagnosed as pustular Psoriasis***. Teriflunomide treatment was stopped and dimethyl fumarate was initiated after 1 month, and the patient remains stable with no recurrence of palmar pustular Psoriasis after 2 years.
	Agirgol et al. [18]	2019	Turkey	54	Female	An MS patient developed a pustular rash on her palms and soles. She had previously taken Dimethyl fumarate but experienced gastrointestinal side effects. She switched to Teriflunomide two months before admission. A dermatological examination revealed ***psoriasiform eruption*** with ***nail abnormalities***. A skin biopsy confirmed the diagnosis. Teriflunomide was discontinued, and treatment with topical corticosteroids and emollient cream led to significant improvement after four months.
	Mancinelli et al. [17]	2017	Italy	55	Female	The patient was referred to a Multiple Sclerosis Centre in 2013 following an episode of acute optic neuritis. She had a familial history of MS and had also undergone surgery for lumbar canal stenosis. After the diagnosis of MS was confirmed, she was treated with Interferon beta-1a, which was discontinued due to lack of efficacy. Teriflunomide was initiated, but the patient reported hair loss and ***progressive nail dystrophy***, leading to the drug being discontinued. The patient's nails showed progressive regrowth and normalization after discontinuation of Teriflunomide.
	Dereure & Camu [2]	2017	France	32	Female	An MS patient with a familial history of Psoriasis developed ***psoriasiform changes in her fingernails*** within five weeks of starting treatment with Teriflunomide. The changes included onycholysis, "salmon" band, and yellowish stain-like discoloration of thickened nail plates. The nail lesions completely vanished within three months of discontinuing Teriflunomide with no relapse eight months later.

Abbreviations: TFN = Teriflunomide; IBD = Inflammatory Bowel Diseases; RRMS = Relapsing-Remitting Multiple Sclerosis.

The type of IBD caused by TFN use was not the same in the two previous reports. In the study by Son et al. [11], the colonoscopic biopsies of an MS patient experiencing severe diarrhea while on TFN, revealed microscopic lesions in the rectosigmoid area with lymphocytic infiltration, indicative of “*lymphocytic colitis”*. On the other hand, Esfahani et al. [3] reported a case, similar to our study, where the patient displayed macroscopic lesions and crypt distortion in the colon, suggestive of “*Crohn's disease”*. Notably, the Esfahani et al. [3] case also involved gastroduodenal ulcers and esophagitis, as seen in upper endoscopy, showing more extensive gastrointestinal involvement than the case in our study.

Regarding the association between TFN and IBD, in 2017, Health Canada reviewed the safety of TFN, focusing on its potential link to colitis. This review was prompted by three post-marketing cases of colitis, with two potentially linked to the drug. Additionally, Health Canada considered nine more cases reported by the manufacturer. However, they couldn't confirm a direct connection between TFN and colitis due to various confounding factors and lack of a clear underlying mechanism. Despite this, Health Canada recommended continued monitoring. Details of the cases weren't specified, but further clinical insights were available from the manufacturer. Notably, a 25-year-old woman developed “*ulcerative colitis”* after a year on TFN, and a 26-year-old man was diagnosed with “*Crohn's disease*” 21 months post-TFN initiation, with symptoms subsiding after stopping the drug but returning upon its reintroduction [3,12].

Moreover, a handful of colitis instances have been linked to leflunomide, which is a precursor to TFN's active form and is employed in managing rheumatoid arthritis (RA) [3,13,14]. In addition, Gugenberger et al.'s study [15] documents a patient with RA undergoing leflunomide treatment who developed subacute colitis. This condition was noted to have a direct correlation with elevated levels of TFN, particularly as the severity of the diarrhea increased with higher TFN levels.

On the other hand, Table 1 provides an overview of five distinct cases of dermatological disorders that correspond with the clinical diagnosis of Psoriasis in individuals who use TFN [2,[16], [17], [18], [19]]. Of these cases, two are characterized by nail lesions [2,17], which bear a striking resemblance to our case report. One case involved pustular skin lesions [16], while the two remaining cases [18,19], manifested a combination of psoriasiform skin pustular and nail lesions occurring concurrently.

During post-marketing monitoring of TFN, data suggested that it might be responsible for the emergence and exacerbation of psoriasis in patients. Similarly, Leflunomide has been linked to the occurrence of psoriasis [19]. The potential ways in which TFN contributes to the development of skin and nail psoriatic lesions are varied and intricate. It's theorized that modifying a segment of the immune system using TFN could result in changes in other segments. This supports the theory that there are numerous pathways involved in psoriasis, with certain pathways being more dominant in different patients [18]. The skin-related adverse effects of TFN, mainly involving hair thinning and loss, suggest its influence on quickly dividing cells such as those in the gastrointestinal tract or epithelium, known for their reduced expression of DHODH. This effect might not be directly attributable to TFN itself but could instead represent a condition known as telogen effluvium. This condition is characterized by a heightened loss of telogen phase hairs and can similarly impact nail health [17]. There is also supporting evidence that TFN may contribute to the emergence or aggravation of psoriasis, a phenomenon similar to the unexpected reactions seen with anti-TNF alpha agents used for non-skin-related conditions. This might involve an unintended activation of inflammation-related pathways, particularly those associated with psoriasis, as a result of pyrimidine synthesis inhibition. Such activation could happen in individuals predisposed to maintaining certain levels of innate or adaptive immunity, possibly due to a malfunctioning feedback mechanism [2,19]. In summary, TFN may induce skin and nail psoriatic lesions through a complex interplay of immune system alterations, impact on rapidly proliferating cells, and paradoxical reactions akin to those seen with anti-TNF alpha agents, indicating the need for careful monitoring of patients on TFN, especially those with a predisposition to psoriasis.

The case presented in this study is noteworthy as it describes a rare occurrence. To the best of our knowledge, the simultaneous occurrence of two Crohn's diseases and Psoriasis resulting from the use of TFN has not been previously reported at all.

## 4 Conclusion

**Fig. 2:**
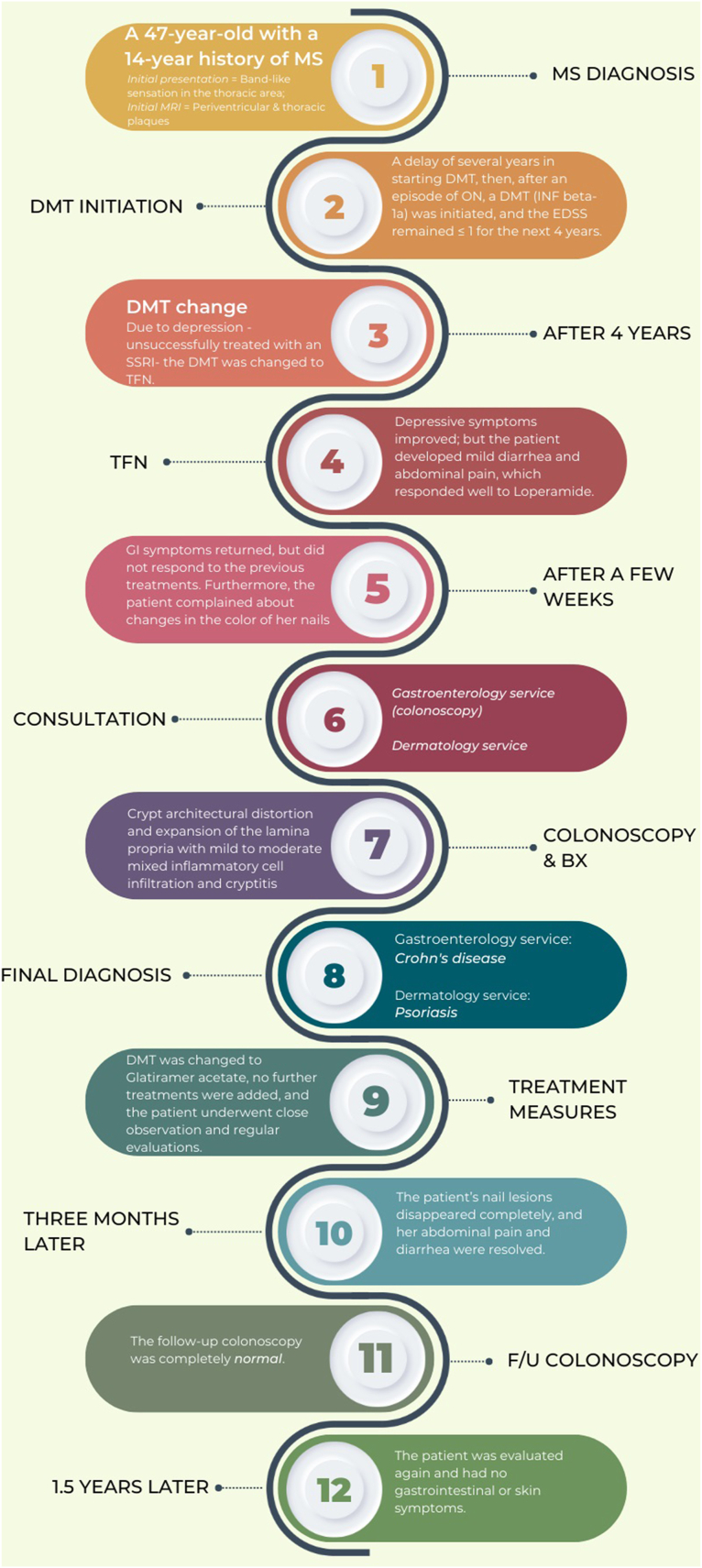
Summary of Case Report; Abbreviations: MS = Multiple Sclerosis; MRI = Magnetic Resonance Imaging; DMT = Disease-Modifying Therapy; ON = Optic neuritis; INF = Interferon; EDSS = Expanded Disability Status Scale; SSRI = Selective Serotonin Reuptake Inhibitor; TFN = Teriflunomide; GI = Gastrointestinal; Bx = Biopsy; F/U = Follow-Up.

This case illustrates a unique presentation of concurrent Crohn's disease and Psoriasis following the administration of TFN in an MS patient (Fig. 2). While TFN is predominantly known for mild side effects, healthcare providers should be vigilant about the potential emergence of severe conditions. The mutual presence of Crohn's and Psoriasis post-TFN intake underscores the intricate interplay of inflammatory pathways in the body and emphasizes the necessity for close monitoring and individualized treatment approaches in MS management.

## Informed consent

*Written informed consent - according to the principles of the Declaration of Helsinki, seventh revision-was obtained from the patient for publication of this case report, any accompanying images, clinical data, and other data included in the manuscript.*

## Funding

None.

## Availability of data and material

Data sharing does not apply to this article as no data sets were generated or analyzed during the current study.

## CRediT authorship contribution statement

**Masoud Ghiasian:** Validation, Supervision, Resources, Data curation, Conceptualization. **Alireza Rastgoo Haghi:** Resources, Investigation, Data curation. **Shiva Borzouei:** Resources, Investigation, Data curation. **Rashed Bawand:** Writing – review & editing, Writing – original draft, Visualization, Data curation.

## Declaration of competing interest

The authors declare that they have no known competing financial interests or personal relationships that could have appeared to influence the work reported in this paper.
